# Ligand-induced conformational changes in a SMALP-encapsulated GPCR.

**DOI:** 10.1016/j.bbamem.2020.183235

**Published:** 2020-06-01

**Authors:** Sarah J. Routledge, Mohammed Jamshad, Haydn A. Little, Yu-Pin Lin, John Simms, Alpesh Thakker, Corinne M. Spickett, Roslyn M. Bill, Tim R. Dafforn, David R. Poyner, Mark Wheatley

**Affiliations:** a Life and Health Sciences, Aston University, Birmingham B4 7ET, UK; b School of Biosciences, University of Birmingham, Birmingham B15 2TT, UK; c Centre for Sport, Exercise and Life Sciences, Alison Gingell Building, Faculty of Health and Life Sciences, Coventry University, Coventry CV1 2DS, UK; d Centre of Membrane Proteins and Receptors (COMPARE), University of Birmingham and University of Nottingham, Midlands, UK

**Keywords:** A_2A_R, adenosine A_2A_ receptor, DMPC, 1,2-dimyristoyl-sn-glycero-3-phosphocholine, ECL, extracellular loop, GPCR, G-protein-coupled receptor, IAEDANS, 5-({2-[(iodoacetyl)amino]ethyl}amino)naphthalene-1-sulfonic acid, ICL, intracellular loop, LC-MS/MS, liquid chromatography with tandem mass spectrometry, *m/z*, mass to charge ratio, PC, phosphatidycholine, PE, phosphatidylethanolamine, PG, phosphatidyglycerol, PS, phosphatidylserine, SMA, styrene maleic acid, SMALP, styrene maleic acid lipid particle, ZM241385, {4-(2-[7-amino-2-(furan-2-yl)[1,2,4]triazolo[1,5-a][1,3,5]triazin-5-yl amino]ethyl)phenol}, GPCR, SMALP, Adenosine receptor, Fluorescence

## Abstract

The adenosine 2A receptor (A_2A_R), a G-protein-coupled receptor (GPCR), was solubilised and purified encapsulated in styrene maleic acid lipid particles (SMALPs). The purified A_2A_R-SMALP was associated with phospholipids characteristic of the plasma membrane of *Pichia pastoris*, the host used for its expression, confirming that the A_2A_R-SMALP encapsulated native lipids. The fluorescence spectrum of the A_2A_R-SMALP showed a characteristic broad emission peak at 330 nm, produced by endogenous Trp residues. The inverse agonist ZM241385 caused 30% increase in fluorescence emission, unusually accompanied by a red-shift in the emission wavelength. The emission spectrum also showed sub-peaks at 321 nm, 335 nm and 350 nm, indicating that individual Trp inhabited different environments following ZM241385 addition. There was no effect of the agonist NECA on the A_2A_R-SMALP fluorescence spectrum. Substitution of two Trp residues by Tyr suggested that ZM241385 affected the environment and mobility of Trp246^6.48^ in TM6 and Trp268^7.33^ at the extracellular face of TM7, causing transition to a more hydrophobic environment. The fluorescent moiety IAEDANS was site-specifically introduced at the intracellular end of TM6 (residue 231^6.33^) to report on the dynamic cytoplasmic face of the A_2A_R. The inverse agonist ZM241385 caused a concentration-dependent increase in fluorescence emission as the IAEDANS moved to a more hydrophobic environment, consistent with closing the G-protein binding crevice. NECA generated only 30% of the effect of ZM241385. This study provides insight into the SMALP environment; encapsulation supported constitutive activity of the A_2A_R and ZM241385-induced conformational transitions but the agonist NECA generated only small effects.

## Highlights

• Conformational changes in the A_2A_R monitored in a nano-scale membrane disc (SMALP).
• Profile of phospholipids in A_2A_R-SMALP similar to the plasma membrane.
• A partially-active conformation of A_2A_R is supported in a SMALP.
• Inverse agonist induced dose-dependent conformational transitions in A_2A_R-SMALP.
• In contrast to inverse agonist, agonist induced only small conformational changes.

## Graphical abstract

**Figure:**
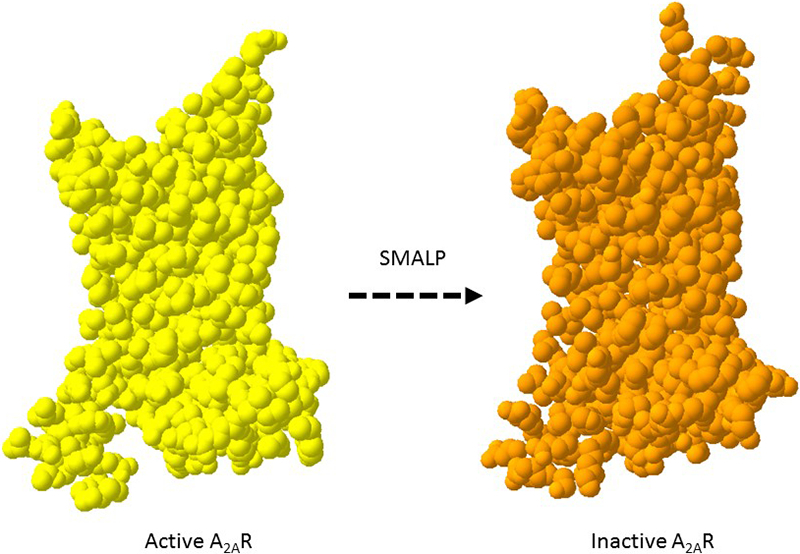

## 1 Introduction

G-protein-coupled receptors (GPCRs) are the largest class of membrane proteins in the human genome (with >800 receptors) and are central to drug discovery programs as they are the therapeutic target for 30–40% of clinically-prescribed drugs [1]. They share a common architecture comprising a bundle of seven transmembrane helices (TMs) [2]. The structural biology of GPCRs is now well established, with crystal structures for >50 different GPCRs currently deposited and over 250 of their complexes with different ligands [3]. This structural information is now being enhanced further by cryo-EM structures of GPCR complexes [4,5]. This extensive structural information has highlighted that GPCRs are highly dynamic proteins and that the receptor conformation is dictated by the structure and efficacy of the bound ligand (full agonist, partial agonist, antagonist or inverse agonist – which inhibits basal signalling). This spectrum of conformations allows these receptors to couple to multiple effectors [6] and for responses to be ‘tuned’, up or down, by allosteric modulators that bind to sites discrete from the orthosteric site used by the natural agonist [7]. This structural knowledge-base has facilitated an understanding of the conserved conformational changes that underpin GPCR activation in general [8]. Nevertheless, it is now recognised that approaches are required that progress beyond static structures to a dynamic understanding. Although solution-based NMR spectroscopy of GPCRs in detergent micelles is starting to address aspects of this [9], it is acknowledged that GPCRs need to be studied in an environment closer to their physiological context within the lipid bilayer of a cell membrane. This is particularly important for investigating GPCRs as they are regulated by the juxtaposition of specific membrane lipids [[10], [11], [12]].

In recent years there has been a growing interest in the use of membrane mimetic systems for studying membrane proteins. The use of poly(styrene-*co*-maleic acid) (SMA) has increasingly been adopted as a strategy for extracting a nanoscale disc of native cell membrane bilayer encapsulated by the SMA polymer, referred to as SMA lipid particle (SMALP) [[13], [14], [15], [16]]. SMA has its limitations in some downstream biological applications. In particular, SMALPs have been shown to be unstable in the presence of divalent cations such as Mg^2+^, with precipitation of the polymer evident at 5 mM Mg^2+^. SMA is also pH sensitive. In an acidic environment (pH values <5.8), the maleic acid groups become protonated causing the polymer to precipitate. This limits the SMALP approach to proteins that function at neutral or basic pHs [17]. Nevertheless, SMALPs have attracted considerable interest for purifying membrane proteins including GPCRs.

We reported the first purification of an active GPCR, in the complete absence of detergent at any stage using the SMALP approach [18,19]; other GPCRs have subsequently been solubilised [20]. The first GPCR-SMALP purified was the the adenosine A_2A_ receptor (A_2A_R). This is a typical GPCR with a well-defined pharmacology. It belongs to a family of four GPCRs (A_1_R, A_2A_R, A_2B_R, A_3_R) that mediate the actions of adenosine and are attractive drug targets [21]. The A_2A_R regulates blood flow to cardiac muscle and regulates the release of the neurotransmitters dopamine and glutamate in the brain, and is well-known as the target for caffeine, which blocks this receptor [22]. It is noteworthy that the A_2A_R has been shown to possess basal activity in the absence of agonist stimulation, referred to as constitutive activity [23]. This receptor is a good choice as a model receptor to investigate the structure/function of a GPCR as there is a wealth of structural information for the A_2A_R with over 40 structures deposited in the Protein Data Bank as listed in the GPCRdb [3]. This includes the conformation of the A_2A_R bound to ligands of different efficacy (full agonist, partial agonist, antagonist, inverse agonist) with corresponding structures of fully active, partially active and inactive, receptor conformations [24].

In this study we investigate ligand-induced conformational changes to the A_2A_R encapsulated in a SMALP using a combination of fluorescence from endogenous Trp residues plus fluorescence from an introduced reporter (IAEDANS) positioned in a dynamic part of the receptor structure, at the cytoplasmic end of TM6. We establish that the A_2A_R adopted at least a partially active conformation in the SMALP and was able to undergo ligand-induced conformational change in response to binding the inverse agonist ZM241385. In contrast, the SMALP-encapsulated A_2A_R exhibited little conformational change in response to the full agonist NECA. This has ramifications for using GPCR-SMALPs as a platform for studying GPCRs.

## 2 Materials and methods

### 2.1 Materials

SMA2000 anhydride was from Cray Valley (UK). ZM241385 {4-(2-[7-amino-2-(furan-2-yl)[1,2,4]triazolo[1,5-a][1,3,5]triazin-5-yl amino]ethyl)phenol} was purchased from Tocris, The mutant constructs [W246Y]A_2A_R and [W268Y]A_2A_R were custom synthesised and cloned into the *Eco*RI-*Not*I sites of the vector pPICZαA by Genscript.

### 2.2 Human A_2A_R expression

An N-terminal His-tagged A_2A_R was expressed in *Pichia pastoris* as described previously [18]. The receptor sequence terminated at Ala316 (Fig. S1) as this construct is degradation resistant [25] and has been used extensively in structural studies, having wild-type pharmacology [26,27]. Prior to SMA-extraction, cells were disrupted following suspension in breaking buffer (50 mM sodium–phosphate buffer, 100 mM NaCl, 5% glycerol, EDTA-free protease inhibitor, pH 7.5, 4 °C) by 3–5 passes using an Avestin Emulsiflex C3 cell-disrupter. Unbroken cells and debris were removed by centrifugation (5000 ×*g*, 10 min, 4 °C). The A_2A_R-expressing membrane fraction was then sedimented (100,000 ×*g*, 60 min, 4 °C) and re-suspended to 80 mg ml^−1^ (wet weight) in extraction buffer (300 mM NaCl, 20 mM HEPES, pH 7.5). Membranes were stored at −80 °C until needed.

### 2.3 Generation of A_2A_R-SMALPs

SMA was prepared from SMA anhydride and used to solubilise A_2A_R from membranes as described previously [16,18]. Briefly, A_2A_R-expressing membrane preparations were thawed on ice, and an equal volume of 2 × SMA buffer (5% *w*/*v* SMA, 300 mM NaCl, 20 mM HEPES, EDTA-free protease inhibitor, pH 7.5) added to yield a final concentration of 40 mg ml^−1^ (wet weight) in 2.5% (*w*/*v*) SMA. Following gentle agitation for 1 h at room temperature, non-solubilised material was removed by centrifugation (100,000 ×*g*, 60 min, 4 °C) to yield a supernatant containing A_2A_R–SMALPs.

### 2.4 Purification of A_2A_R-SMALPs

All purification steps were carried out at 4 °C. The A_2A_R–SMALP supernatant was incubated with ~1 ml Ni^2+^-NTA resin, overnight on an end-over-end rotator. The column was washed with 20 column volumes (cv) of wash buffer (300 mM NaCl, 20 mM HEPES, 25 mM imidazole, EDTA-free protease inhibitor pH 7.5). Elution of A_2A_R–SMALP was achieved with 10 cv of elution buffer (300 mM NaCl, 20 mM HEPES, 250 mM imidazole, EDTA-free protease inhibitor pH 7.5). Elution fractions were pooled, buffer-exchanged into assay buffer (300 mM NaCl, 20 mM HEPES, pH 7.5) and concentrated using spin-concentrators (10 kDa cut-off). Concentrations of purified A_2A_R-SMALP were determined using SDS-PAGE and densiometric analysis against protein standards in ImageJ. Final concentrations ranged between 0.2 and 1 mg ml^−1^.

### 2.5 IAEDANS labelling

Ala231^6.33^ of the A_2A_R was mutated to Cys using the QuikChange site-directed mutagenesis kit (Stratagene, Cambridge, UK) according to the manufacturer's instructions. The oligonucleotide primers 5′-G-AAA-GAG-GTT-CAC-**TGT**-GCT-AAG-TCC-TTG-3′ (sense) and 5′-CAA-GGA-CTT-AGC-**ACA**-GTG-AAC-CTC-TTT-C-3′ (antisense) were used, with appropriate base changes shown in bold. All receptor constructs were confirmed by automated fluorescent sequencing. For labelling by IAEDANS (5-({2-[(iodoacetyl)amino]ethyl}amino)naphthalene-1-sulfonic acid), 0.05 mg of [A231C]A_2A_R-SMALP in phosphate buffer (50 mM NaH_2_PO_4_, 300 mM NaCl, pH 8.0) was added to 3 μl of 5 mM IAEDANS in DMSO (10-fold excess of IAEDANS) in a total reaction volume of 100 μl and incubated for 3 h at 20 °C with gentle rotation. Samples were then prepared using a Vivaspin 500 centrifugal concentrator (10,000 MWCO; 15,000 ×*g*, 2 min), washed with 3 × 200 μl phosphate buffer then centrifuged as above to reduce sample volume to 100 μl. Labelling of ‘empty SMALPs’ (DMPC-SMALPs) containing the lipid DMPC but lacking A_2A_R was performed using the same protocol.

### 2.6 Fluorescence measurements

Fluorescence measurements were made using a PTI QuantaMaster 300 fluorimeter using continuous Xe arc excitation. Samples were contained in a 0.3 cm quartz fluorescence cuvette (Starna Scientific). Emission spectra were obtained by exciting the protein at 295 nm and 340 nm for tryptophan and IAEDANS respectively. Excitation slit widths of 0.2 mm (±0.8 nm) and emission slit widths of 0.8 mm (±3.2 nm) were used for all measurements with a scan speed of 500 nm/min. Emission spectra were measured between 300 and 400 nm for tryptophan fluorescence experiments and 425–625 nm for IAEDANS. For determination of Stern-Volmer constants, acrylamide was titrated into the cuvette up to a final concentration of 2 M. At each titration point the emission intensity of the tryptophan was measured. The resulting intensity data were adjusted to take into account the effect of diluting the A_2A_R-SMALP, then plotted to provide a Stern-Volmer constant using the equation F_0_/F_−__1_ = 1 + κ_q_τ_0_[Q], where F_0_ is the intensity of fluorescence in the absence of quencher, F_−__1_ is the intensity with the quencher, present at concentration [Q], τ_0_ is the lifetime of the excited state and κ_q_ is the Stern-Volmer constant.

### 2.7 Lipid extraction

Total lipid was extracted from *P. pastoris* cells using the method described by Spickett et al. 2001 [28]. Shake flask cultures of 25 ml were set up and approximately 5 ml culture harvested 48 h post-induction. The culture was centrifuged at 10,000 ×*g* and the supernatant discarded. The cell pellets weighed approximately 50 mg and 0.5 ml methanol at 50 °C was added before incubating in a sonicating water bath for 15 min. 0.5 ml chloroform was then added and the cells sonicated for a further 15 min. 0.5 ml of 0.88% KCl was added and the mixture was vortexed. The cells were centrifuged at 10,000 ×*g* for 2 min to separate the organic and aqueous layers. The organic (lower) layer was transferred to a fresh tube and dried under a stream of nitrogen gas. The lipid extracts were stored at −80 °C until analysis. For lipid extraction from A_2A_R–SMALPs, approximately 50 ml of cell culture was used, and the resulting A_2A_R–SMALP preparation after purification according to Section 2.4 was subjected to methanol-chloroform extraction as described above for total lipids.

### 2.8 Liquid chromatography with tandem mass spectrometry (LC-MS/MS)

Phospholipid extracts of *Pichia pastoris* membranes were reconstituted in 200 μl 1:1 methanol: chloroform (v/v) and diluted (typically 1/1000) in 20% isopropyl alcohol in acetonitrile. Aliquots (10 μl) were injected via an autosampler onto a ACE 3-SIL HILIC column (150 × 3.1 mm, Hichrom, UK) and separated using a U3000 HPLC system controlled by Chromeleon software (ThermoFisher, Hemel Hempstead, UK). The HPLC was interfaced to a 5600 TripleTOF mass spectrometer (ABSciex, Warrington, UK) via a TurboSpray® ion source. Elution was achieved by a multi-step gradient as follows: 0–1 min held at 5% B; 1–5 min to 8% B; 5–10 min to 15% B; 10–13 min held at 15% B; 13–23 min to 35% B; 23–28 min held at 35% B; 28–29 min to 5% B; 29–45 min held at 5% B, where solvent A was 20% isopropyl alcohol in acetonitrile and solvent B was 20% isopropyl alcohol in 20 mM aqueous ammonium formate. The flow rate was set to 300 μl/min throughout. The source temperature was set at 350 °C; the spray voltage was 5500 V; the declustering potential was set to 50 V for all scans; nitrogen was used as the curtain gas and nebulising gas with flow rates set to 35 AU and 26 AU respectively. Survey scan MS data were acquired by electrospray ionization in positive mode from 400 to 1200 Da in high resolution mode for 500 ms. Information dependent data acquisition (IDA) was used to collect MS/MS data based on following criteria: the 4 most intense ions with +1 charge and a minimum intensity of 250 cps were chosen for analysis, using dynamic exclusion for 20 s after 2 occurrences and a fixed collision energy setting of 47 eV.

### 2.9 Molecular modelling

Models of [W246Y]A_2A_R and [W268Y]A_2A_R were built in Modeller [29] using the A_2A_R with bound ZM241385 (PDB ID: 3EML) as a template. 1000 models were generated for each mutation and scored using the Modeller objective scoring function. The best models were subsequently relaxed using Rosetta [30]. Ligands were docked and scored using PLANTS under the default settings [31].

### 2.10 Residue numbering

The Ballesteros-Weinstein nomenclature system for family A GPCR residues is employed throughout this article, indicated by a superscript number [32]. This provides the unique position of each residue with two numbers; its transmembrane helix plus its position relative to the most conserved residue (ascribed the number 50) in that helix across all family A GPCRs. This numbering system allows direct comparison of residues between different GPCRs.

## 3 Results

### 3.1 Tryptophan fluorescence of A_2A_R-SMALP

**Fig. 1:**
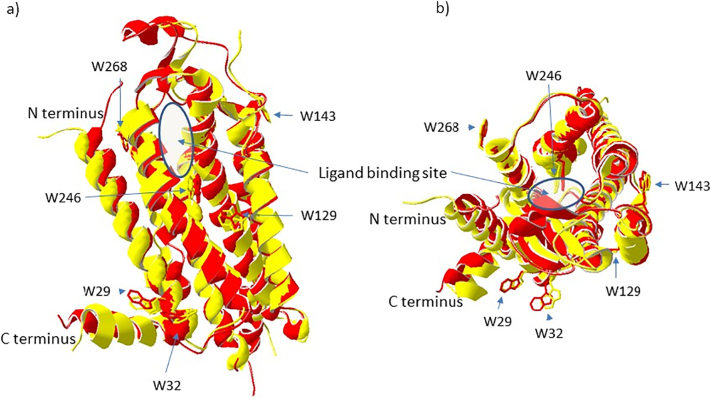
Location of the Trp residues in the A_2A_R. The structure of ZM241385 (inverse agonist)-bound A_2A_R (yellow; PDB ID: 3EML) is aligned to the structure of the NECA (agonist)-bound A_2A_R (red; PDB ID: 2YDV) with the Trp residues indicated, viewed through the plane of the membrane (panel a) and from above (panel b).

The A_2A_R-SMALPs were generated using SMA (2.5% *w*/*v*) to extract the receptors from A_2A_R-expressing membranes into SMALPs, followed by purification of the A_2A_R-SMALPs as previously described [18]. Each sample of purified A_2A_R-SMALP was tested for specific binding of the A_2A_R radio-ligand [^3^H]ZM241385 to ensure that it was pharmacologically active. The wild-type A_2A_R has seven tryptophan residues but the C-terminally truncated construct used in the current study has only six (Supplentary Fig. 1a). Of these, Trp29^1.55^ and Trp32^1.58^ are at the bottom of TM1, at the junction with the first intracellular loop (ICL1). Trp129^4.50^ is in TM4. Trp143^4.64^ is at the junction between TM4 and extracellular loop 2 (ECL2), Trp246^6.48^ is in TM6 and Trp268^7.33^ is at the extracellular face of TM7 at the junction with ECL3 (Fig. 1).

**Fig. 2:**
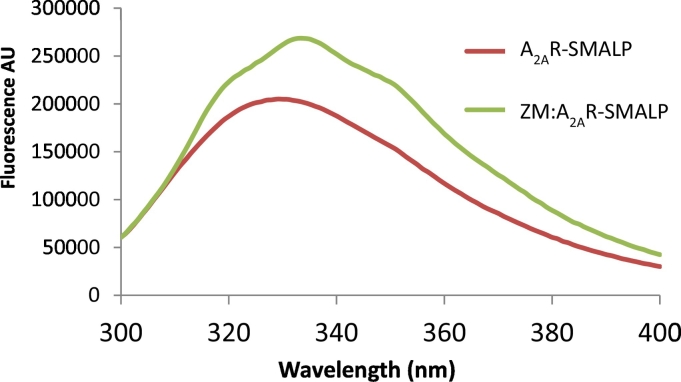
Fluorescence of A_2A_R-SMALP. Fluorescence spectrum in the absence (A_2A_R-SMALP) and presence (ZM:A_2A_R-SMALP) of ZM241385 (1 μM).

The initial stage of the investigation was to determine the Trp fluorescence spectrum of the apo A_2A_R-SMALP, in the absence of any ligand (Fig. 2). The spectrum exhibited a broad peak (reflecting the multiple Trp in the receptor) with a maximum of ~330 nm, indicating that the Trp residues are located in relatively non-polar environments. This is consistent with the buried positions of the Trp residues within the receptor structure, as seen in the crystal structures of the receptor (Fig.1). The fluorescence of ZM241385 at 330 nm is 2550 AU, <1.5% of that of the receptor, indicating that the changes seen in Fig. 2 are due to the A_2A_R. We also determined the fluorescence spectrum of the A_2A_R after solubilisation with the detergent *n*-dodecyl-*β*-D-maltopyranoside (data not shown); it also had a single peak at approximately 330 nm, suggesting that the overall folding of the protein was similar in the SMALP and detergent.

### 3.2 Ligand-induced changes in tryptophan fluorescence

To investigate the effect on the Trp fluorescence spectrum of ligands binding to the A_2A_R encapsulated in a SMALP, two ligands possessing different pharmacological properties (efficacy) were employed; i) ZM241385, an A_2A_R-selective inverse agonist which suppresses receptor activation [25] and ii) NECA, an agonist that fully activates the receptor. Binding of ZM241385 increased the Trp fluorescence A_2A_R-SMALP markedly (30%) compared to the unoccupied receptor (Fig. 2). In contrast there was no change in the fluorescence with NECA (data not shown). The 30% increase in the Trp fluorescence of the ZM241385:A_2A_R-SMALP complex compared to the apo A_2A_R reveals a ZM241385-induced conformational change in the receptor structure upon ligand binding. Intriguingly, the increase in fluorescence intensity was accompanied by a red shift. This is unusual as it would be expected that an increase in intensity (interpreted as a movement of Trp residues into a less polar environment) should be accompanied by a corresponding blue shift. However, close examination of the spectrum of the ZM241385-bound conformation offers some insight. The apo A_2A_R-SMALP spectrum shows only a single broad peak at 330 nm indicating that the Trp residues are either in similar environments or that their peaks are broad and overlap to a large degree. It can be seen in Fig. 2, that ZM241385 binding induced the single peak to resolve into three clear sub-peaks at approximately 321 nm, 335 nm and 350 nm. These peaks are likely to represent individual Trp residues affected by the change in local structure induced by ZM241385 binding, resulting in a significant change in their spectral characteristics. It is therefore possible that the unusual redshift observed upon ZM241385 binding is the net result of complex changes in the fluorescence arising from multiple Trp transitions in the protein.

### 3.3 Changes in Trp fluorescence correlate with ZM241385 binding to A_2A_R-SMALP

**Fig. 3:**
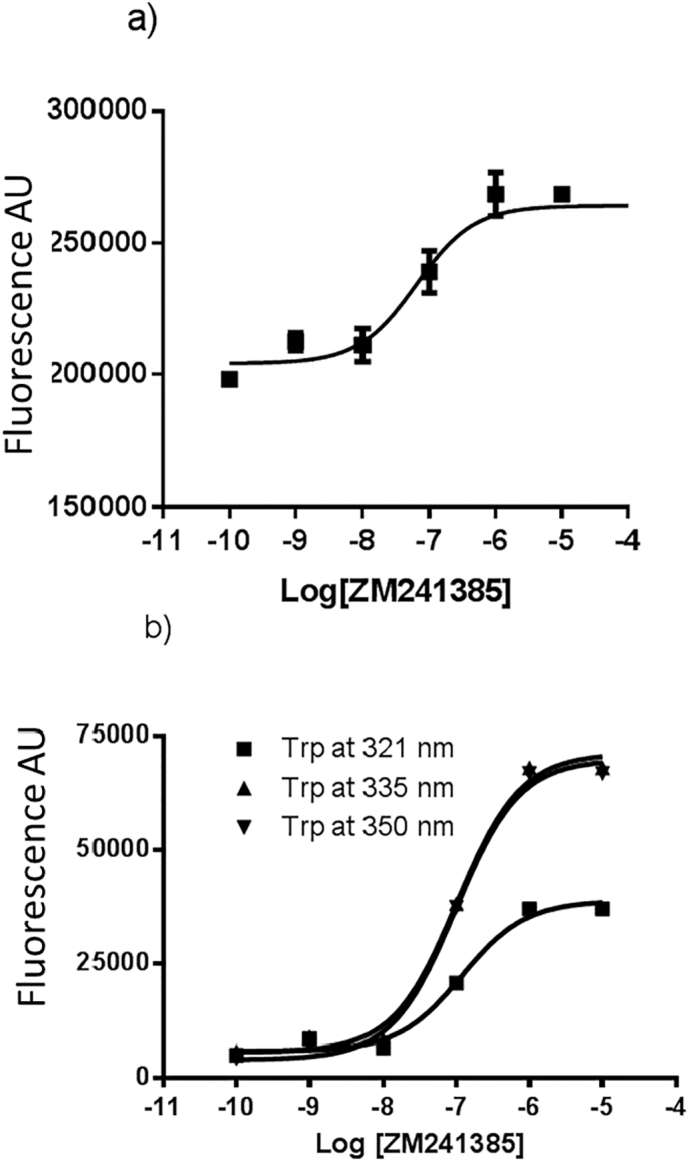
ZM241385-induced changes in fluorescence of A_2A_R-SMALP. Panel a; the dose-dependent effect of ZM241385 binding on the overall fluorescence. Panel b; the dose-dependent effect of ZM241385 binding on the individual fluorescence peaks at 321 nm, 335 nm and 350 nm.

The concentration-dependence of changes in the Trp fluorescence observed when ZM241385 bound was determined. The effect of ZM241385 binding on the overall Trp fluorescence is presented in Fig. 3a. This exhibits a pEC_50_ = 7.2 ± 0.22 (*n* = 3) which is comparable to the corresponding value for ZM241385 binding to A_2A_R-SMALP (7.79 ± 0.14, n = 3) and binding to A_2A_R in whole membranes (7.95 ± 0.45, n = 3). As the fluorescence spectrum of the ZM241385-bound state exhibited three distinct sub-peaks of fluorescence (at 321 nm, 335 nm and 350 nm), the response of each of these to increasing concentrations of ZM241385 was analysed. The ZM241385 concentration-response curves at 321 nm, 335 nm and 350 nm gave similar pEC_50_ values of 6.93 ± 0.14, 7.00 ± 0.10 and 7.01 ± 0.10 respectively (Fig. 3b). This indicates that each of the putative Trp residues that correspond to these peaks are responding to the same binding event.

### 3.4 Solvent accessibility of Trp residues in apo and ligand-bound states

**Fig. 4:**
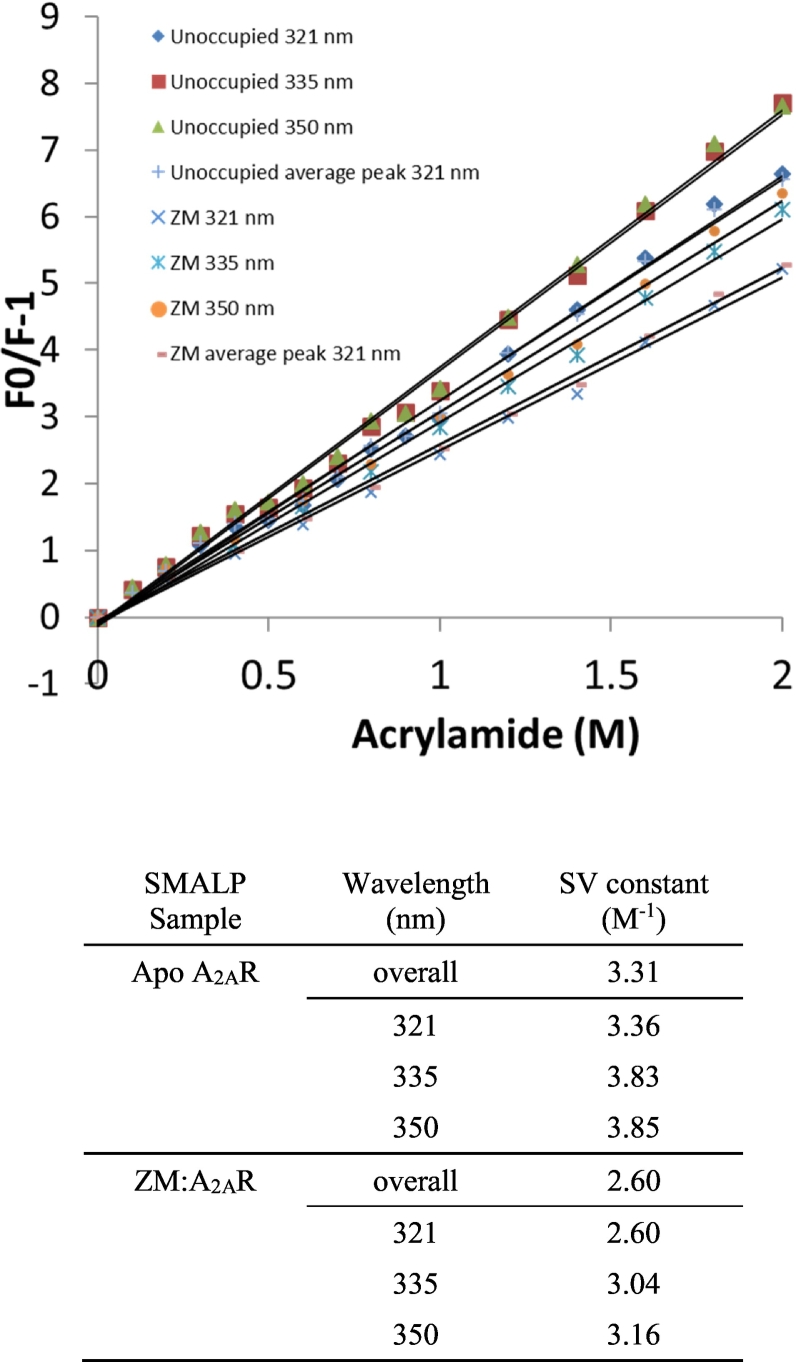
Stern-Volmer quenching data. Upper panel; quenching data for A_2A_R-SMALP and ZM:A_2A_R-SMALP. Lower panel; calculated SV constants for overall fluorescence and for individual sub-peaks.

Stern-Volmer (SV) quenching experiments were performed to compare the solvent accessibility of the Trp residues in the unoccupied apo A_2A_R-SMALP versus the ZM241385-bound state. Acrylamide was used as the fluorescence quenching agent, as it has the advantage of being uncharged thereby avoiding any potential charge-related problems with the polymer component of the SMALP. A full emission spectrum was determined at each acrylamide concentration, so that an SV constant could be calculated for the overall fluorescent species (by producing an SV curve from an average of all data). In addition, SV constants were also calculated for each of the Trp peaks (321 nm, 335 nm, 350 nm) resolved in the ZM241385-bound state (Fig. 4a). In all cases, the binding of ZM241385 to the A_2A_R-SMALP decreased the SV constant (Fig. 4b), indicating that the solvent accessibility of the Trp residues in the apo A_2A_R decreased upon ZM241385 binding. This agrees well with our earlier observation that the overall fluorescence intensity of the A_2A_R-SMALP increased when ZM241385 occupied the receptor (Fig. 2), indicating that the Trp residues moved to a less polar environment.

Interestingly, there was a difference between the SV constants calculated at different positions in the spectrum (corresponding to the three peaks observed in the ZM241385-bound state. Fig. 4b). The SV constants for 335 nm and 350 nm were very similar (3.04 and 3.16 respectively), suggesting that Trp residues generating these peaks have similar solvent accessibilities. In contrast, the 321 nm peak has a much lower SV value (SV = 2.60) indicating a decreased solvent exposure compared to 335 nm and 350 nm. This latter result correlates well with the blue-shifted nature of the 321 nm peak.

### 3.5 Measurement of fluorescence anisotropy changes upon ZM241385 binding

Fluorescence anisotropy can be used to determine changes in fluorophore flexibility. Depending upon the fluorescence lifetime of the fluorophore, this can include detecting changes in the tumbling time of the assembly in solution, as well as changes in the motions of the fluorophore within the context of the assembly. We have already shown in this study that the binding of ZM241385 to the A_2A_R-SMALP altered the environment and solvent accessibility of one or more of the Trp residues. It is also likely that ZM241385 binding altered the steric freedom of these residues in the receptor. It is however, unlikely that the overall tumbling of the A_2A_R-SMALP would be affected significantly by ZM241385 binding to the SMALP-encapsulated A_2A_R.

Measurements of the fluorescence anisotropy of A_2A_R-SMALP showed that binding of ZM241385 produced a clear increase in anisotropy (apo A_2A_R-SMALP, 0.1044 ± 0.0027; ZM241385-bound A_2A_R-SMALP, 0.1341 ± 0.0020, values are mean ± s.e.m., *n* = 3, *p* < .001). This indicated that the Trp residues are less mobile in the ZM241385:A_2A_R-SMALP complex than in the unoccupied apo A_2A_R-SMALP. Again, this agrees well with other data in this study (Fig. 2, Fig. 3, Fig. 4) showing that Trp residues in the ZM241385-bound state are in a less polar, less solvent-exposed and potentially more sterically-hindered, environment.

### 3.6 Analysis of Trp-mutated A_2A_R constructs

Individual Trp residues in the A_2A_R were mutated to Tyr in an attempt to deconvolute the contributions made to the overall fluorescence spectrum by at least some of the Trp residues. Trp246^6.48^ and Trp268^7.33^ were selected for Tyr substitution as it was reasoned that they were most likely to report on A_2A_R conformational changes, as TM6 and TM7 are dynamic helices in GPCRs generally and are linked to GPCR activation [8]. Both [W246Y]A_2A_R and [W268Y]A_2A_R were well-expressed, although the affinity for ZM241385 for these mutant receptors was reduced by 6-fold and 27-fold, respectively ([W246Y]A_2A_R - pIC_50_ 6.99 ± 0.46; [W268Y]A_2A_R - pIC_50_ 6.36 ± 0.17; wild-type A_2A_R pIC_50_ 7.79 ± 0.14). This was not unexpected as Trp246^6.48^ forms part of the binding site for ZM241385 [33] and Trp268^7.33^ may influence the local conformation at the extracellular face of TM7/ECL3, controlling ligand access to the binding pocket. Despite this, it was still possible to measure changes in the fluorescence spectrum caused by ZM241385 binding to the mutant constructs, suggesting that the overall structure of the protein was maintained. This was further supported by computational molecular modelling which revealed that the predicted structures of [W246Y]A_2A_R and [W268Y]A_2A_R were virtually superimposable on that of WT A_2A_R, with RMS deviations of <0.01Å (Supplementary Fig. 2), suggesting that there was no gross disruption of folding.

**Fig. 5:**
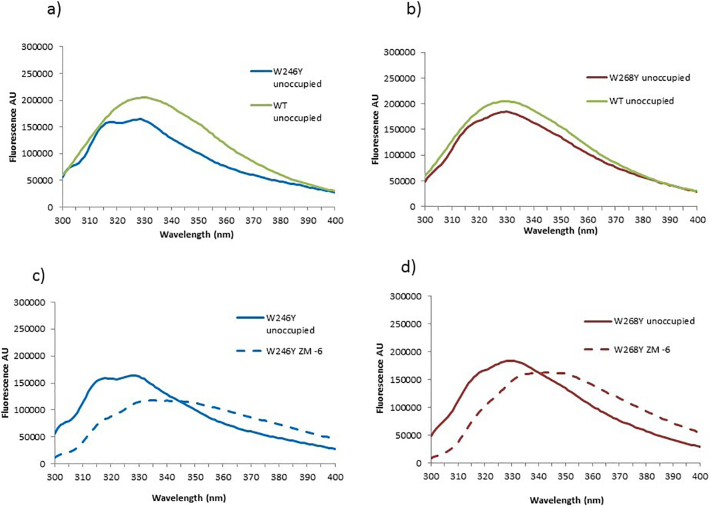
Effect of substitution of individual Trp residues on A_2A_R-SMALP fluorescence. Panel a; A_2A_R-SMALP and [W246Y]A_2A_R-SMALP in the absence of ZM241385. Panel b; A_2A_R-SMALP and [W268Y]A_2A_R-SMALP in the absence of ZM241385. Panel c; A_2A_R-SMALP and [W246Y]A_2A_R-SMALP with ZM241385 (1 μM) bound. Panel d; A_2A_R-SMALP and [W268Y]A_2A_R-SMALP with ZM241385 (1 μM) bound.

In the absence of ZM241385, the spectrum of [W246Y]A_2A_R-SMALP showed two clear peaks, one at 329 nm (like the WT A_2A_R-SMALP) and a second at 315 nm (Fig. 5). Given that the protein can still bind ZM241385, the appearance of this second peak is likely to result from the removal of the fluorescence from Trp246^6.48^, probably centred close to 320 nm, which would obscure the fluorescence at 315 nm in the WT A_2A_R. The spectrum of [W268Y]A_2A_R in the absence of ZM241385 also showed two peaks, again one at 329 nm (like the WT) plus a second peak at 315 nm (Fig. 5). The relative intensities are different to those observed for [W246Y]A_2A_R-SMALP but again these data are indicative of the mutated Trp having an emission between 315 and 329 nm.

Addition of ZM241385 to both mutant receptors led to a significant decrease in fluorescence and accompanying red shift (Fig. 5). The red shift agreed with that seen in the WT A_2A_R, however the intensity change is completely different (although a red shift is more usually seen with an intensity decrease). This suggested that the substituted Trp residues contributed an increase in fluorescence upon ZM241385 binding to the WT A_2A_R. Their substitution means that these fluorescence decreases were caused by one or more of the remaining Trp residues moving to a more polar environment in response to ZM241385 binding. The corollary of this, is that both Trp246^6.48^ and Trp268^7.33^ moved into a less polar environment upon binding ZM241385.

### 3.7 Analysis of IAEDANS-labelled A_2A_R

**Fig. 6:**
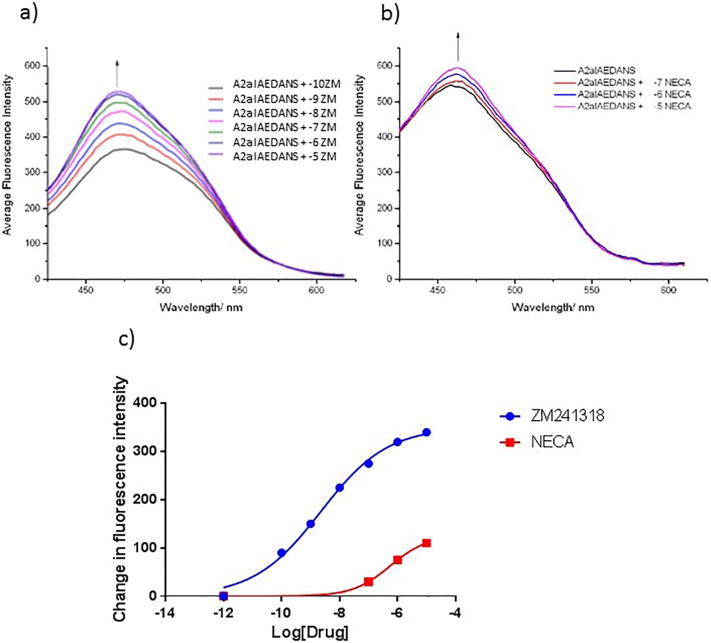
Ligand-induced changes in IAEDANS fluorescence. The effect on fluorescence intensity of exposure to ZM241385 (10^−10^ M – 10^−5^ M; panel a) or NECA (10^−7^ M – 10^−5^ M; panel b) at the concentrations indicated was determined for A_2A_R-SMALP labelled with IAEDANS at residue-231. Panel c; Concentration-response curves for the fluorescence intensity changes induced by ZM241385 or NECA.

To investigate ligand-induced conformational changes in the cytoplasmic face of the A_2A_R, a fluorescent reporter (IAEDANS) was introduced at residue 6.33, located at the cytoplasmic end of TM6. A fluorescent moiety at this locus within the receptor architecture is well-placed to report on the dynamic changes that are known to occur at the intracellular face of the helical bundle during receptor activation. In particular, the large outward movement of the cytoplasmic end of TM6 away from the helical bundle that creates the binding crevice in the receptor for G-protein docking [8]. The IAEDANS reagent used in the current study reacted with thiol groups, so the strategy employed involved introducing a Cys at residue 231^6.33^ followed by derivatisation of the A231C mutant construct by IAEDANS. The A_2A_R used in this study contained fourteen Cys residues (Supplementary Fig. 1b), so it might be assumed that this would prevent such a strategy. However, upon closer examination, it can be seen that the eight Cys residues in the extracellular loops contribute to disulphide bonds (Cys71^2.69^ - Cys159^5.20^; Cys74^3.22^ - Cys146^4.67^; Cys77^3.25^ - Cys166^5.27^ and Cys259^6.61^ - Cys262^6.64^) so will not react with IAEDANS. The remaining six Cys residues (Cys28^1.54^; Cys82^3.30^; Cys128^4.49^; Cys185^5.46^; Cys245^6.47^; Cys254^6.56^) are located in the TMs (Supplementary Fig. 1b), so are buried to varying degrees and therefore would be only poorly accessible to the IAEDANS. This was confirmed in control experiments that established the fluorescence of the WT A_2A_R-SMALP (with Ala231^6.33^) after IAEDANS labelling was identical to that of ‘empty SMALP’ formed of lipid only and devoid of any A_2A_R (Supplementary Fig. 3). Mutation of A231^6.33^ did not alter the binding capability of the receptor (Supplementary Fig. 4) and following IAEDANS-labelling of Cys231^6.33^, a robust fluorescent signal was detected (Fig. 6). The effect of ligand binding on the fluorescence intensity of the IAEDANS-labelled A_2A_R-SMALP was investigated. Binding of the inverse agonist ZM241385 to A_2A_R-SMALP generated a marked dose-dependent increase in fluorescence (Fig. 6a) with a pEC_50_ of 8.74 ± 0.32 (*n* = 3), indicating that ZM241385 binding drove the IAEDANS to move to a more hydrophobic environment. In contrast, the agonist NECA produced only a 9% increase in fluorescence (Fig. 6b) that was nevertheless dose-dependent (Fig. 6c) with a pEC_50_ of 6.37 ± 0.21 (n = 3).

### 3.8 Analysis of lipid composition

**Fig. 7:**
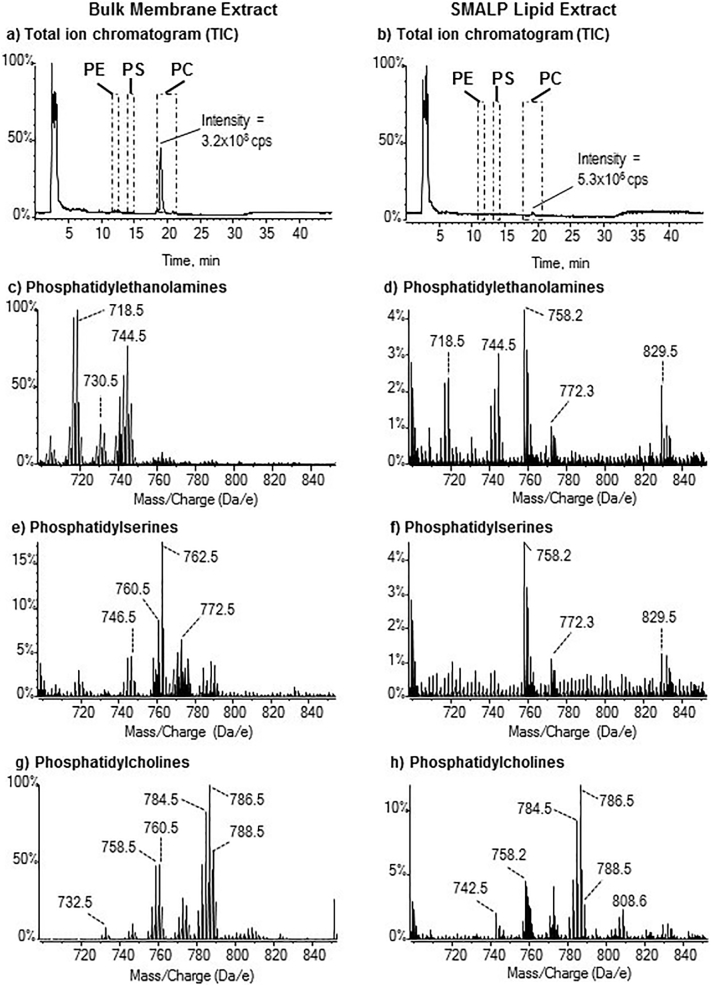
Comparison of the phospholipid profiles of bulk membrane lipids, and SMALP-extracted lipids, from *P. pastoris*. Different classes of phospholipids were separated by HILIC and analysed by electrospray MS in positive ion mode. Data were summed across each of the elution regions in the TICs shown in panels a and b to generate the spectra for each class shown in the panels c-h. The y-axis shows the % intensity in counts per second (cps) normalized to 100% for the most intense peak in the spectrum across the mass range analysed. For each class of phospholipid, the y-axes of the bulk membrane and SMALP spectra are linked to allow a relative comparison of intensity. Note that phospholipid signals give *m/z* values with decimal 0.5 and signals with *m/z* values ending 0.2 or 0.3 are background contaminants. Identifications of the individual phospholipids are given in Table 1.

To confirm that the use of SMALPs for membrane protein extraction allows the local lipid environment to be retained, LC-MS/MS was used to characterize the A_2A_R-associated phospholipids extracted from *P. pastoris* membranes using SMALPs, in comparison with the total membrane phospholipids (Fig. 7). The phospholipid species in SMALP extracts were identified based on the accurate mass and in some cases additionally their fragmentation pattern, and are listed in Table 1. The phospholipid elution profile on the HILIC column was determined by running standard mixtures of phosphatidylethanolamine (PE), phosphatidylserine (PS) and phosphatidylcholine (PC), which allowed these lipid classes to be identified in chromatograms of total membrane and SMALP extracts (Fig. 7a and b, respectively). Although the amount of phospholipid extracted using SMALPs was, not surprisingly, significantly less than that obtained from total cell extracts, PE and PC could clearly be identified (Fig. 7c and d; Fig. 7g and h). The levels of PS were low even in total membrane extracts, and in SMALP extracts they were essentially below the limit of detection (Fig. 7e and f). As the phospholipid levels in SMALP extracts were low, signals from background contaminant ions in the clusters centred on *m/z* 758.2, 772.3 and 829.5 are prominent, especially in Fig. 7f. Overall, whilst there was some evidence that the ratio of poly-unsaturated to mono-unsaturated PC species was slightly higher in SMALP extracts (e.g. *m/z* 758.5 vs 760.5, *m/z* 786.5 vs 788.5 and higher relative intensity of *m/z* 808.5), the profile of SMALP-extracted A_2A_R-associated phospholipid appeared to be generally similar to the total membrane.

**Table 1:** Phospholipid species detected by high resolution LC-MS/MS in SMALP-extracted *P. pastoris* samples.

Experimental m/z	Retention time (min)	Molecular formula	Calculated m/z	Error in ppm	Phospholipid species
*Glycerophosphocholines* ([*MH*]^+^)					
732.5528	19.36	C_40_H_79_NO_8_P	732.5543	2.1	GPC (32:1)
744.5530	19.27	C_41_H_79_NO_8_P	744.5543	1.8	GPC (16:1/17:1)^a^
746.5673	19.31	C_41_H_81_NO_8_P	746.5700	3.6	GPC (33:1)
754.5373	19.27	C_42_H_77_NO_8_P	754.5387	1.8	GPC (34:4)
756.5529	19.24	C_42_H_79_NO_8_P	756.5543	1.9	GPC (34:3)
758.5667	19.25	C_42_H_81_NO_8_P	758.5694	3.7	GPC (16:1/18:1)^a^
760.5828	19.27	C_42_H_83_NO_8_P	760.5851	3.7	GPC (34:1)
768.5510	19.22	C_43_H_79_NO_8_P	768.5538	3.6	GPC (18:3/17:1)^a^
770.5667	19.20	C_43_H_81_NO_8_P	770.5694	3.5	GPC (35:3)
772.5844	19.20	C_43_H_83_NO_8_P	772.5851	1.6	GPC (18:1/17:1)^a^
780.5526	19.22	C_44_H_79_NO_8_P	780.5538	2.2	GPC (18:3/18:2)^a^
782.5667	19.18	C_44_H_81_NO_8_P	782.5695	3.6	GPC (18:2/18:2)^a^
784.5823	19.14	C_44_H_83_NO_8_P	784.5851	3.3	GPC (36:3)
786.5979	19.14	C_44_H_85_NO_8_P	786.6008	3.7	GPC (18:1/18:1)^a^

*Glycerophosphoethanolamines ([MH]*^*+*^*)*					
690.5063	12.43	C_37_H_73_NO_8_P	690.5074	1.6	GPE 32:1)
704.5217	12.54	C_38_H_75_NO_8_P	704.5230	1.9	GPE (33:1)
716.5220	12.73	C_39_H_75_NO_8_P	716.5230	1.4	GPE (34:2)
718.5370	12.68	C_39_H_75_NO_8_P	718.5387	2.3	GPE (34:1)
738.5059	12.47	C_41_H_73_NO_8_P	738.5074	2.0	GPE (36:5)
742.5369	12.67	C_41_H_77_NO_8_P	742.5387	2.4	GPE (36:3)
744.5528	12.70	C_41_H_79_NO_8_P	744.5543	2.1	GPE (18:0/18:2)^a^
764.5201	12.65	C_43_H_75_NO_8_P	764.5230	3.8	GPE (38:6)
766.5348	12.65	C_43_H_77_NO_8_P	766.5387	5.1	GPE (38:5)

*Glycerophosphoserines* ([*MH*]^+^)					
762.526	14.12	C_40_H_77_NO_10_P	762.5285	3.3	GPS (34:1)

For other phospholipid species the total fatty acyl chain length: double bonds are given.

a Fatty acyl chain composition determined by observation of fatty acyl ions in MS/MS spectra.

## 4 Discussion

To fully understand the structure and function of a membrane protein it is necessary to extract it from the lipid bilayer for purification. This usually requires detergents to disrupt the membrane but generally leads to protein instability as the lipids of the bilayer are stripped away [34]. In recent years there has been a large increase in the use of SMA to extract a wide range of membrane proteins encapsulated in SMALPs. This preserves the native membrane environment of the protein albeit on a nanoscale, and improves stability. We established previously that the A_2A_R could be purified encapsulated in a SMALP with retention of wild-type ligand binding capability [18]. It is well-established that GPCRs can form dimers and that heterodimerization can modulate GPCR signalling. Such interaction is maintained within a SMALP, as a tetrameric (2 + 2) complex of ghrelin receptor (GHSR) and dopamine receptor (D2R), isolated by SMALP-solubilisation of reconstituted lipid vesicles, retained GHSR modulation of D2R signalling [35]. The aim of the current study was to investigate the conformational changes induced by binding ligands to the A_2A_R when it is encapsulated in a SMALP.

We were able to demonstrate that SMALP-encapsulated A_2A_R contained phospholipids typical of the plasma membrane, including PCs and PEs. It has been reported previously that interactions of some GPCRs, e.g. neurotensin receptor 1, with G-proteins are dependent on a native-like lipid environment containing PEs [36], although the β_2_-adrenergic receptor showed higher activity in the presence of phosphatidylglycerols (PGs) than PEs [10]. Other GPCRs have been reported to require cholesterol [11,37] and the active state of the A_2A_R is stabilised by the binding of phosphatidylinositol 4,5-bisphosphate [12]. Although the plasma membrane of *P. pastoris* differs from that of mammalian cells, notably in substitution of cholesterol by ergosterol, the major classes of phospholipids are similar and the A_2A_R was pharmacologically active. Phosphatidylinositols and inositol ceramides are minor, though important, membrane components and in the present study were below the detection limit of the method; hence it is not possible to comment on their contribution to the activity observed. However, proteins within SMALPs can be exchanged into detergent or amphipol [38] or into vesicles of alternative phospholipid composition [39] and SMALP phospholipids can also be exchanged [40], which in the future offers alternative approaches for improving GPCR activity following isolation in SMALPs.

In its native membrane, the A_2A_R exhibits considerable constitutive activity in the absence of agonist, which can account for over 50% of the agonist-stimulated activity [23] and this can be reduced by inverse agonists such as ZM241385 [41]. It is well-established that activation of GPCRs involves conformational changes that are conserved throughout the receptor family [8]. In particular, there is a large (10 - 14 Å) movement of the cytoplasmic end of TM6 away from the helical bundle that opens a cleft in the cytoplasmic face of the receptor for G-protein docking [42]. The fluorescent moiety IAEDANS introduced at residue 231^6.33^, at the cytoplasmic face of TM6, is therefore well-placed to report on the dynamic changes that occur on the intracellular face of the A_2A_R during activation. Indeed, a fluorescent reporter located at the corresponding locus in the β_2_-AR (A271^6,33^) was used previously to monitor activation of the β_2_-AR [43].

The IAEDANS data in the current study show that SMALP-solubilised A_2A_R retains substantial elements of the constitutively active conformation of A_2A_R existing in the native membrane environment. Binding of ZM241385 caused a substantial dose-dependent increase in fluorescence emission. The simplest explanation of this is that the IAEDANS probe moved into a more hydrophobic environment. This is what would be expected if the cytoplasmic end of TM6, in the absence of the inverse agonist, was part of an open conformation to accommodate binding of G_sα_ and binding of ZM241385 closed the G_sα_ binding crevice. Such conformational transitions and movement of TM6 between active and inactive A_2A_R are consistent with the corresponding crystal structures [44]. Kinetic data indicate that the binding of ZM241385 is biphasic, consistent with conformational changes of the receptor [45]. In contrast, the agonist NECA caused very little change in IAEDANS fluorescence emission.

The fluorescence of endogenous Trp residues is a powerful technique to study the conformation of native proteins. Trp are important residues in their own right. Their large size, coupled with the ability to take part in hydrophobic and columbic interactions means that they often act as switches or pivots during conformational changes. There are a number of highly conserved Trp residues in GPCRs, including Trp^6.48^ in TM6, referred to as the “rotamer toggle switch” [46]. An agonist-induced shift of this conserved Trp^6.48^ promotes the outward tilt of the intracellular part of TM6 required for G-protein coupling described above. It has already been noted that IAEDANS fluorescence emission was affected by ZM241385 binding and ZM241385 also caused changes in the environments of the Trp residues in the SMALP-solubilised A_2A_R. The agonist NECA however, did not affect the Trp fluorescence. The predominant change upon ZM241385 binding involved an increase in fluorescence emission and the appearance of three distinct peaks in the fluorescence spectrum at 321 nm, 335 nm and 350 nm. Interestingly, two of these peaks (321 nm, 335 nm) have wavelengths that are not far from those observed in the spectra of the unliganded mutant constructs [W246^6.48^Y]A_2A_R-SMALP and [W268^7.33^Y]A_2A_R-SMALP (peaks at 315 nm and 329 nm). This confirms that in each conformation there are Trp residues present that maintain similar spectroscopy.

Analysis of the Trp/Tyr substitution mutants suggests that Trp246^6.48^ and Trp268^7.33^ are responsible for the increase in the fluorescence emission observed when ZM241385 binds to the WT. These Trp residues are not located near to the cytoplasmic face of the A_2A_R and thus report on different regions from that measured by the IAEDANS at residue 231^6.33^. For Trp246^6.48^ in TM6, two mechanisms may contribute to the change in fluorescence. The antagonist may trigger a conformational change in the A_2A_R-SMALP that moves the residue to a more hydrophobic environment. However, binding of ZM241385 could also reduce the solvent accessibility of Trp246^6.48^ as it is located in the ZM241385 binding pocket where it forms a hydrophobic contact with the furan ring of ZM241385 [33], along with adjacent residues [47]. Both mechanisms could decrease the rotation of the residue, consistent with the measured reduction in anisotropy and hence fluorophore mobility. However, as NECA also binds close to Trp264^6.48^ and yet does not change Trp fluorescence, it seems a conformational change in the receptor is the most likely explanation.

Our observation that the inverse agonist ZM241385, which inhibits activation, caused the ‘rotamer toggle switch’ Trp246^6.48^ of the A_2A_R-SMALP to move to a more hydrophobic environment is the exact opposite effect to that accompanying photo-activation of rhodopsin. Photo-activation of rhodopsin causes the corresponding residue (Trp265^6.48^) to transition to a more hydrophilic environment [48]. These two observations are consistent with respect to linking the nature of the Trp^6.48^ environment and the activation state of the GPCR.

Trp268^7.33^ is at the extracellular face of TM7, on the boundary between the membrane and the extracellular aqueous medium. ZM241385 binds in an extended conformation perpendicular to the plane of the membrane. Nevertheless, the changes seen here are unlikely to be due to direct contact with ZM241385 with Trp268^7.33^, so probably reflect changes in receptor conformation, although crystal structures reveal that ZM241385 does make hydrophobic interactions with other residues at the extracellular face of TM7 (L267^7.32^ and M270^7.35^) [49,50]. There is also evidence for movement of ECL3 during activation of the A_2A_R with molecular dynamic studies suggesting that compaction of the extracellular domain of the A_2A_R underlies the positive allosteric effects of divalent cations as they bridge across acidic residues in ECLs [51]. Even a small movement of the indole head-group of Trp268^7.33^ towards the membrane, induced by ZM241385 binding, would be expected to significantly reduce its polarity and could potentially reduce its mobility. Another factor that could be relevant to changes in the environment of residues within the TM helical bundle are the ordered water molecules within the receptor structure [49,52]. Whilst the changes seen with Trp246^6.48^ and Trp268^7.33^ dominate the spectrum, their substitution by Tyr unmasks changes due to other Trp residues having greater solvent accessibility. Caution is needed in interpreting this as the Trp/Tyr mutations have some effect on ZM242385 binding. Nevertheless, despite this caveat, it is at least plausible that in the WT receptor some of the Trp residues on, or close to, the intracellular or extracellular loops will have greater solvent exposure following antagonist binding, thus experiencing an increase in polarity.

In conclusion, the SMALP-solubilised A_2A_R adopted a conformation that retained the native constitutive activity. Using fluorescence changes from endogenous Trp residues and an introduced IAEDANS, we established that the receptor was able to undergo ligand-induced conformational changes with the SMALP environment in response to binding the inverse agonist ZM241385. In contrast, the SMALP-encapsulated A_2A_R exhibited little conformational change in response to the full agonist NECA.

## Declaration of competing interest

The authors declare that they have no known competing financial interests or personal relationships that could have appeared to influence the work reported in this paper.

## Appendix A Supplementary data

Supplementary figures Image 1
