# Supplementary material for: Ligand-induced conformational changes in a SMALP-encapsulated GPCR
Source: Biochim Biophys Acta Biomembr. 2020 Jun 1;1862(6):183235. doi: 10.1016/j.bbamem.2020.183235 (PMC7156913; doi:10.1016/j.bbamem.2020.183235)
Supplement: Supplementary file 1 — Supplementary figures [file mmc1.docx]

**Supplementary Figures.**

a)

b)

**Supplementary Fig. 1.** Schematic diagram of the A_2A_R.

Panel a; Trp residues are shown as grey circles. Panel b; Cys residues are shown as coloured circles in the extracellular loops and as grey circles in the TM helices. Disulphide bonds are indicated as per the crystal structure (PDB ID: 3EML).


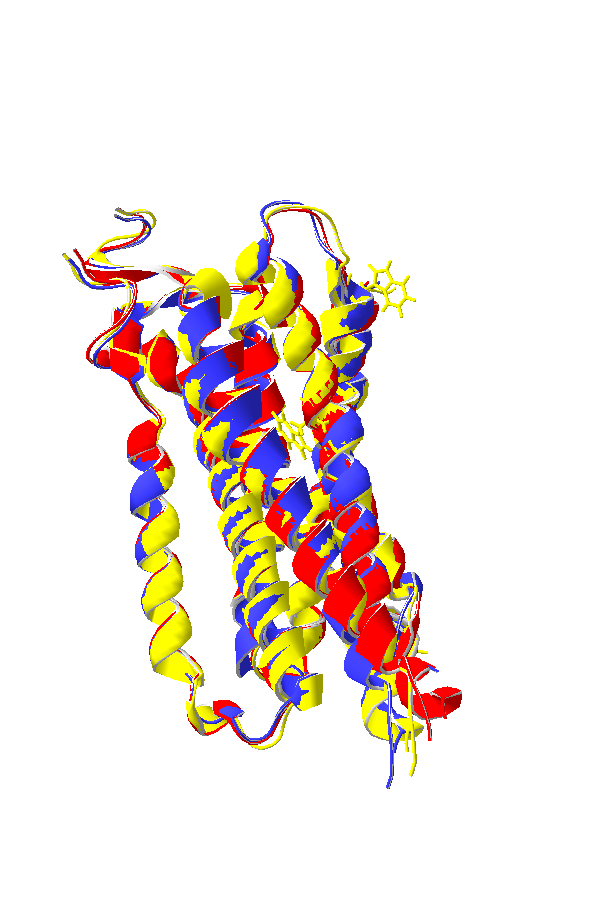


**Supplementary Fig. 2.** Comparison of A_2A_R structures.

The structure of WT A_2A_R bound to ZM241385 (yellow) is aligned with the predicted structures of [W246Y]A_2A_R (red) and [W268Y]A_2A_R (blue) from computational molecular modelling. RMS deviations less than 0.01A.


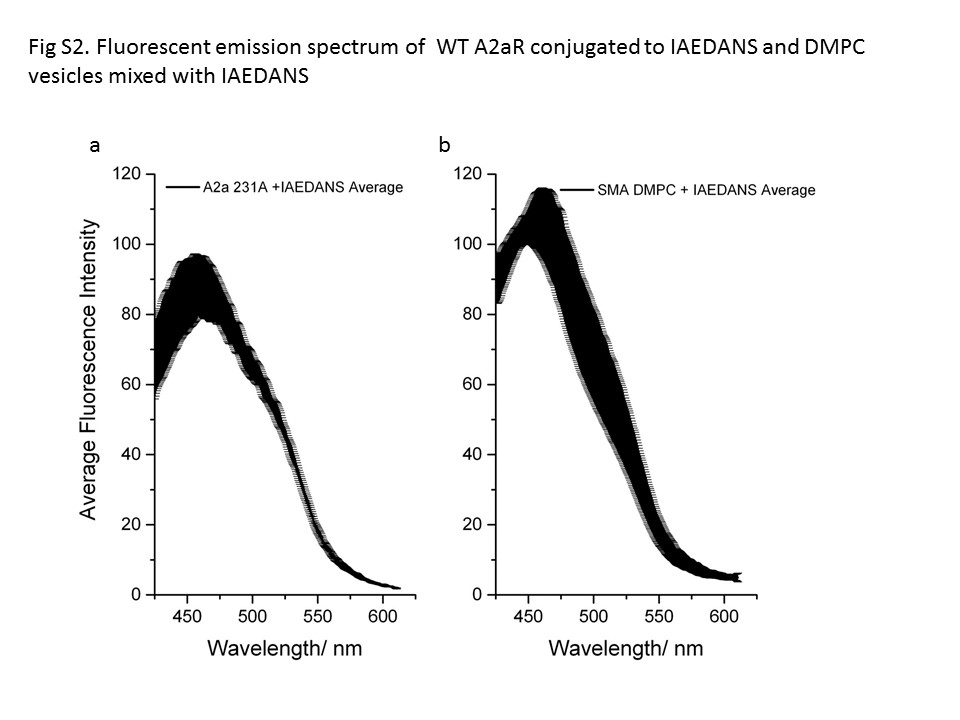


**Supplementary Fig. 3**. Fluorescent emission spectrum of IAEDANS.

Panel a; IAEDANS fluorescent emission spectrum of WT A_2A_R (Ala231). Panel b; IAEDANS fluorescent emission spectrum of ‘empty SMALPs’ containing only the lipid DMPC.

**Supplementary Fig. 4**. Mutation of Ala231^6.33^ does not affect ZM241385 binding.

Binding curves for ZM241385 binding to wild-type (■) and mutant (▼) A_2A_R.
